# GPT-4.1 and Llama 3.3 70 fail to detect clinically relevant errors in radiology reports in zero-shot evaluation

**DOI:** 10.1007/s00330-026-12697-z

**Published:** 2026-06-19

**Authors:** Tugba Akinci D’Antonoli, Lisa C. Adams, Jannik Lübberstedt, Markus M. Graf, Christian J. Mertens, Felix Busch, Sebastian Ziegelmayer, Marcus R. Makowski, Keno Bressem, Ina Luiken

**Affiliations:** 1 Division of Diagnostic and Interventional Neuroradiology, Department of Radiology, University Hospital Basel, Basel, Switzerland; 2 Department of Pediatric Radiology, University Children’s Hospital Basel, Basel, Switzerland; 3 Institute of Diagnostic and Interventional Radiology, TUM University Hospital, TUM School of Medicine and Health, Technical University of Munich, Munich, Germany; 4 Department of Cardiovascular Radiology and Nuclear Medicine, German Heart Center Munich, TUM University Hospital, Munich, Germany

**Keywords:** Large language models, Artificial intelligence, Errors

## Abstract

**Objectives:**

To evaluate whether GPT-4.1 and Llama 3.3 70B, large language models (LLMs) assessed in zero-shot, baseline configurations, detect and categorize clinically consequential errors across types that range from pattern-based to reasoning-dependent.

**Materials and methods:**

Two hundred fifty-six radiology reports encompassing CT (*n* = 104), MRI (*n* = 104), and X-ray (*n* = 48) studies across multiple anatomical regions were retrospectively analyzed. For each original report, four variants (*n* = 1024) were generated, each incorporating one of four predefined error types: E1, anatomical mislabeling that could cause wrong-site actions; E2, physiologically impossible or nonsensical findings; E3, diagnostic inconsistencies that affect staging or diagnosis; E4, inappropriate recommendations. The evaluated models were GPT‑4.1 04-14) and Llama 3.3 70B, both used without domain-specific training or prompt optimization to assess baseline model performance.

**Results:**

Model performance revealed a systematic hierarchy governed by error type and imaging modality. Physiologically impossible errors (E2) showed the lowest performance: 46.2% (CT) and 33.7% (MRI) for GPT-4.1, compared with 32.7% and 25.0% for Llama 3.3, respectively. Overall success for GPT-4.1 on E2 was 16.3% (CT), 8.7% (MRI), and 12.5% (X-ray). Mislabeling errors (E1) were detected in 49.0% by GPT‑4.1 and 33.7% by Llama 3.3 for MRI. Best performance occurred for inappropriate recommendations (E4), with GPT‑4.1 achieving 85.4% detection in X-ray with high classification accuracy.

**Conclusion:**

The evaluation framework and benchmark dataset provide a methodology for assessing LLM performance on clinically significant errors. Applied to GPT-4.1 and Llama 3.3 70B in zero-shot settings, the framework reveals a performance gap between pattern-based and reasoning-dependent error detection that warrants investigation across additional models and optimization strategies.

**Key Points:**

***Question*** *LLMs are increasingly used for quality assurance of radiology reports, but whether their linguistic competence translates into the detection of clinically significant errors remains unclear*.

***Findings*** *Error detection was type-dependent, both GPT-4.1 and Llama 3.3 70B performed poorly on physiologic and anatomical errors, but better on inappropriate recommendations*.

***Clinical relevance*** *LLMs failed to detect most clinically consequential errors in radiology reports, especially physiologically impossible statements that trained radiologists would rapidly identify*.

**Graphical Abstract:**

**Figure:**
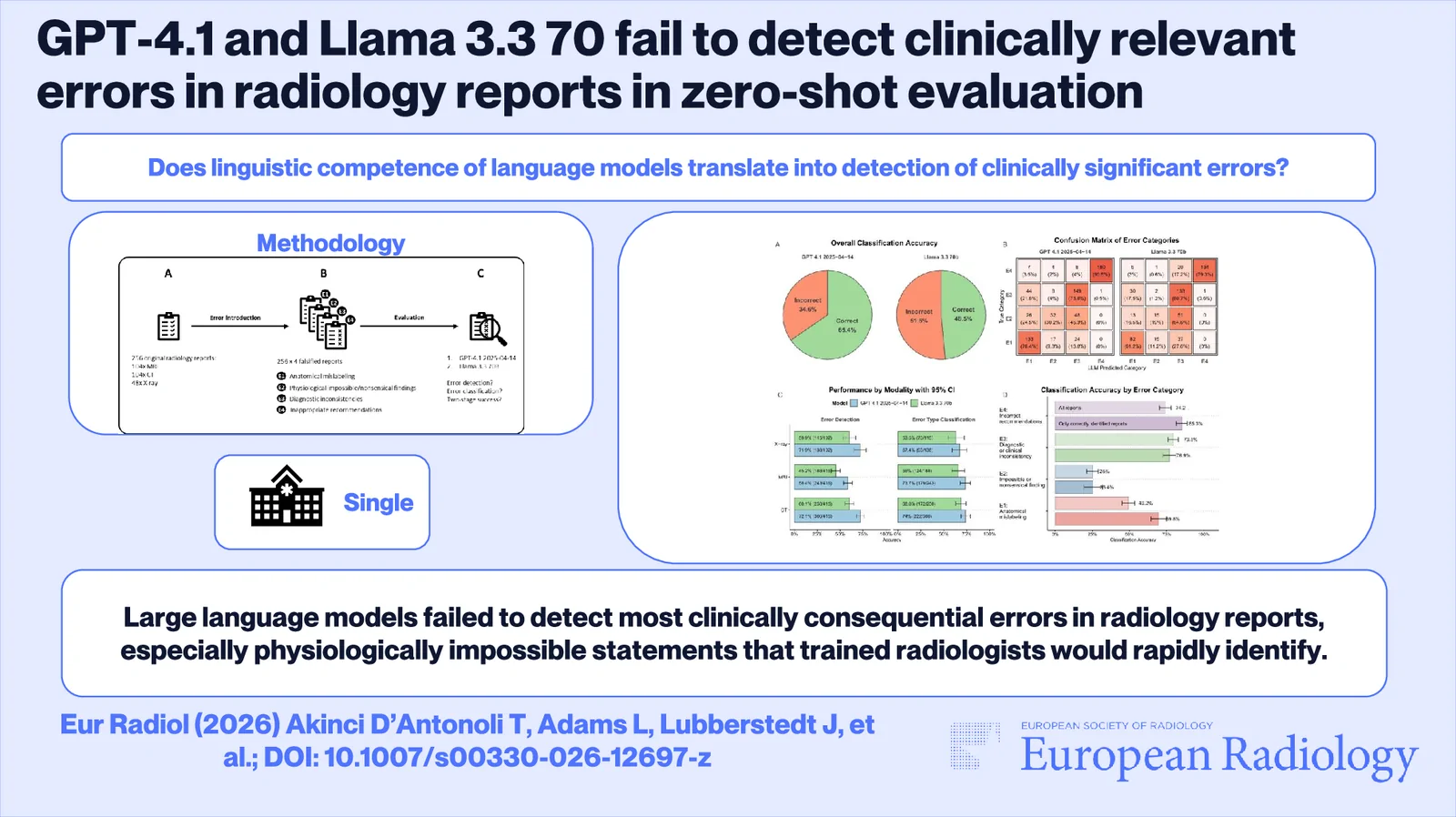

## Introduction

Workload in radiology continues to rise with consistently increasing numbers of radiography, computed tomography (CT), and magnetic resonance imaging (MRI) examinations that are performed annually worldwide. This increasing clinical workload is associated with greater susceptibility to errors in radiology reports [1]. Driven by their impressive performance in general language tasks, large language models (LLMs) have also attracted interest for radiology tasks [2–4]. They have recently been evaluated on a wide range of applications, including, but not limited to, information extraction, structuring, summarization, decision support, and error detection [5–12].

However, high performance in linguistic fluency does not necessarily equate to clinical safety. As LLMs are increasingly considered for integration into clinical workflows, a key concern is their ability to identify and interpret medically significant errors, especially mistakes that may otherwise go unnoticed and result in diagnostic delays or patient harm [13]. Moreover, LLMs could be misused for cyberattacks by injecting false medical data into radiology reports to profit from unnecessary tests or follow-ups [14].

Studies have underscored this gap, emphasizing that while LLMs can mimic expert phrasing, they often lack the domain-specific reasoning, e.g., potentially leading patients to draw harmful conclusions when used to simplify radiology reports [15–17]. More recently, the evaluation of LLMs for detecting clinically relevant errors in radiology reports has become an area of increasing interest, reflecting the increasing reliance on automated systems for medical documentation and diagnostic support, with findings suggesting a disconnect between generative fluency and factual reliability [18, 19]. Furthermore, while models with multimodal and retrieval-augmented generation capabilities have an improved contextual understanding, they have not resolved fundamental limitations in logical consistency and clinical prioritization [15]. Few studies have benchmarked these tools across diverse modalities, and to our knowledge, among them, only a few have systematically evaluated Llama models in this context [12].

A critical distinction exists between errors that follow formulaic patterns, such as contradictory recommendations, and errors that require domain-specific reasoning, such as recognizing physiological impossibilities. LLMs may perform well at detecting the former while failing to identify the latter, yet most evaluations have not systematically tested this distinction.

In this study, we propose an evaluation framework for systematically assessing LLM error detection in radiology reports and apply it to GPT-4.1 and Llama 3.3 70B as an initial benchmark. Using 256 radiology reports across CT, MRI, and X-ray, each modified with four categories of clinically relevant errors (1024 total), we evaluate whether LLM performance is governed by error type, testing the hypothesis that a gap exists between pattern-based and reasoning-dependent error detection. The evaluation framework and dataset are made publicly available to facilitate benchmarking of future models.

## Materials and methods

### Study design

**Fig. 1:**
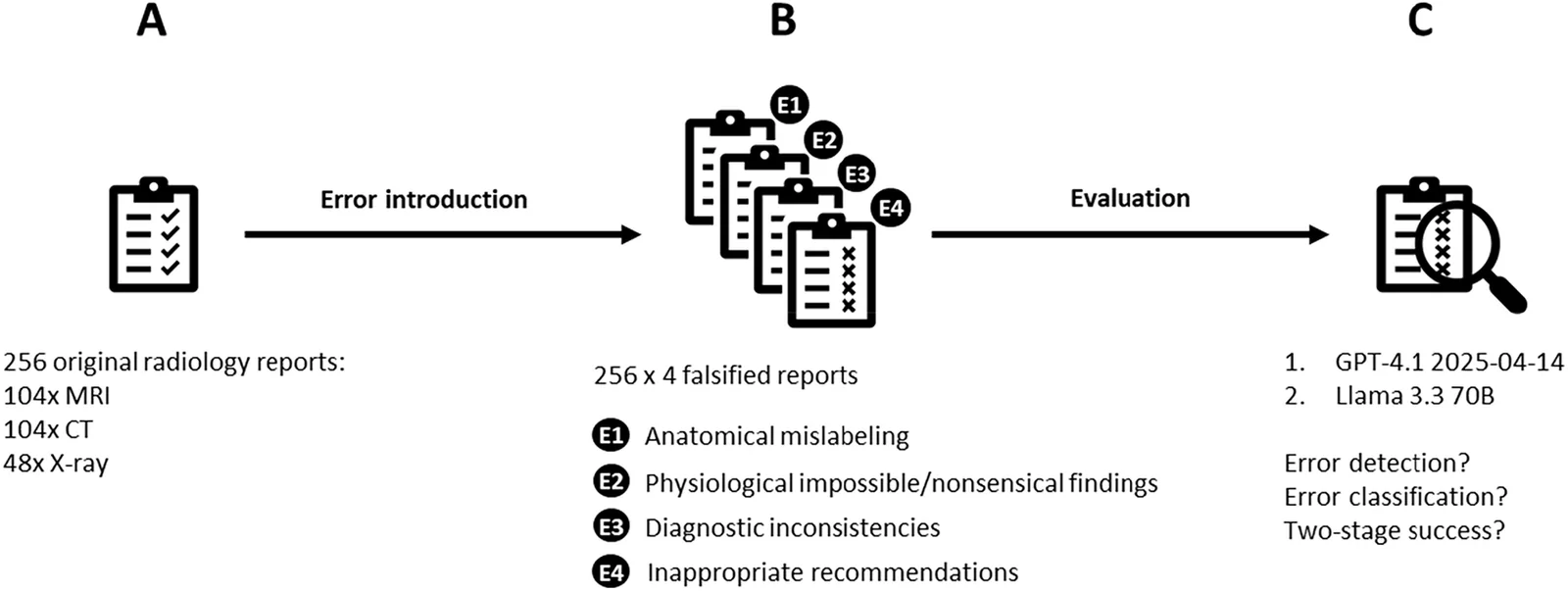
Study Overview. **A** A set of 256 original radiology reports was compiled, including 104 MRI, 104 CT, and 48 X-ray reports. These reports served as the baseline reference. **B** Each report was quadrupled, with each copy containing a single, deliberately introduced error from one of four predefined error categories (E1: anatomical mislabeling, E2: physiologically impossible or nonsensical findings, E3: diagnostic inconsistencies, E4: Inappropriate recommendations). This process generated a total of 1024 falsified reports. **C** The complete set of 1024 falsified reports was evaluated for error detection (presence or absence of an error), followed by error classification into one of the four categories and a two-stage success rate, where detection and classification were correct, using GPT-4.1 and Llama 3.3 70b

This retrospective analysis was conducted with ethics approval (2024-590-S-CB, and informed consent was waived due to the synthetic nature of the data). We evaluated structured radiology reports created to test clinically relevant error detection capabilities. A schematic illustration of the study workflow is provided in Fig. 1.

### Dataset construction

Reports were retrospectively selected from anonymized routine clinical examinations at a single academic medical center. In total, 256 radiology reports distributed across three imaging modalities were included. These reports were retrospectively selected from anonymized routine clinical examinations conducted at a single academic medical center. CT examinations (*n* = 104) included thoracic (*n* = 28), abdominal (*n* = 28), cranial (*n* = 24), and musculoskeletal (*n* = 24) studies. MRI examinations (*n* = 104) comprised brain (*n* = 28), spine (*n* = 28), abdominal (*n* = 24), and musculoskeletal (*n* = 24) regions. Radiographic studies (*n* = 48) included chest (*n* = 24) and musculoskeletal (*n* = 24) examinations.

For each report, we created four report variants, each containing different types of errors (*n* = 1024). Each error (E) variant was labeled 1 through 4 (e.g., E1). The errors were created and injected into the reports by one board-certified radiologist (T.A.D.) and a 4th-year radiology resident (I.L.). E1 (anatomical mislabeling) placed pathology in incorrect anatomical locations that could lead to inappropriate procedures. E2 (physiologically impossible or nonsensical findings) described findings that violate fundamental biological or physical principles. E3 (diagnostic inconsistencies) contained contradictory statements affecting diagnosis and staging. E4 (inappropriate recommendations) suggested management contradicting the described findings (Table 1). The original dataset was in German and translated into English using large language model–based translation tools (i.e., DeepL). All translations were manually reviewed and verified for accuracy by the authors during the error injection process. The resulting English version served as the base dataset for all subsequent error variations. The dataset is publicly available at https://doi.org/10.5281/zenodo.17529445.

**Table 1:** Examples from each error category, along with their clinical relevance

Error type	Original	Error	Clinical relevance
E1 (anatomical mislabeling)	‘A 12 mm lesion is identified in the right frontal lobe.’	‘A 12 mm lesion is identified in the left frontal lobe.’	Could lead to a wrong-site biopsy or surgical planning.
E2 (physiologically impossible)	‘Calcifications in the falx cerebri, pineal gland, and choroid plexus’	‘Metal calcifications in the falx cerebri, pineal gland, and choroid plexus.’	Describes a physical impossibility (metal calcification is physiologically impossible) that any radiologist would immediately recognize.
E3 (diagnostic inconsistency)	‘Stable oncological findings of an NSCLC (diagnosed 10/2019) under Capmatinib (since 10/2023).’	‘Progressive oncological findings of an NSCLC (diagnosed 10/2019) under Capmatinib (since 10/2023).’	Impression is inconsistent with the findings.
E4 (inappropriate recommendation)	‘No acute cardiopulmonary findings. No follow-up necessary.’	‘No acute cardiopulmonary findings. Recommend urgent CT pulmonary angiography.’	Unnecessary follow-up for normal findings.

Two board-certified radiologists (L.C.A. and K.K.B.) independently verified that each error represented a plausible mistake with potential clinical consequences. Disagreements were resolved through consensus discussion.

### Model evaluation

**Fig. 2:**
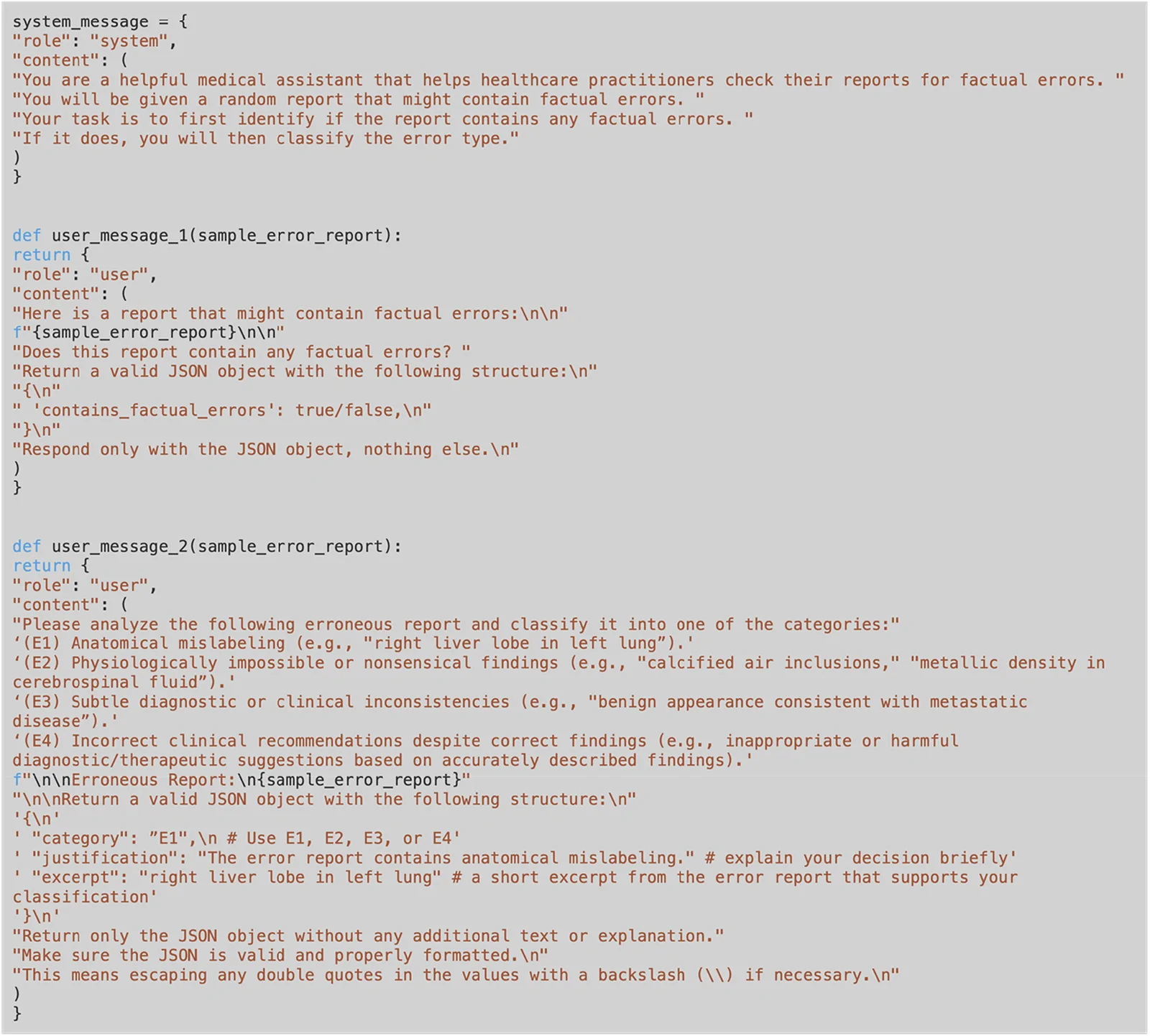
Standardized prompt requesting error detection (binary decision) followed by error classification (four categories) received by both models

We evaluated GPT-4.1 (release 2025-04-14, OpenAI) and Llama 3.3 70b instruct (Meta), which were selected as representative models from the two most widely deployed LLM families in medical AI research, enabling comparison between proprietary and open-source architectures. The inclusion of an open-source model is particularly relevant for clinical deployment scenarios where data privacy regulations may necessitate local hosting and preclude the use of cloud-based proprietary APIs. Each model received standardized prompts requesting error detection (binary decision) followed by error classification (four categories) (Fig. 2).

Models were accessed via the OpenAI API (GPT-4.1) and Together AI API (Llama 3.3 70b at half precision without quantization). Each radiology report was processed as an independent API call. For all API calls temperature was set to 0, and all other parameters were kept at their default values. The models were evaluated in a zero-shot setting without training examples, medical context, or prompt optimization, to assess baseline model performance. The exact prompts used are provided in the Supplementary Material. A single evaluation run was performed for each model.

### Statistical analysis

Primary endpoints included error detection accuracy (proportion of reports with errors correctly identified as containing errors), error classification accuracy (proportion of detected errors correctly categorized into E1-E4 categories), and two-stage success rate (proportion of reports where both detection and classification were correct). Performance metrics were calculated overall and stratified by imaging modality (CT, MRI, and X-ray) and error category (E1–E4). Differences in proportions between error categories, modalities, and models were assessed using chi-squared tests, with Fisher’s exact test applied where expected cell counts were below 5. Statistical significance was defined as *p* < 0.05. All reported *p*-values are two-sided. Descriptive statistics with proportions and counts were used to summarize model performance. The 95% confidence intervals (CI) were calculated using the percentile bootstrap method (10,000 bootstrap samples), providing distribution-free CI for proportion estimates. A formal a priori power analysis was not performed, as the dataset size was determined by clinical availability. However, with 1024 error-containing reports and observed effect sizes exceeding 25 percentage points for the primary comparisons (E4 vs E2 detection), the study provides sufficient statistical power to detect the magnitude of differences reported, as confirmed by non-overlapping 95% bootstrap CI. All analyses were performed using R version 4.3.1.

## Results

### Primary performance metrics

Both models showed intermediate primary performance metrics using the percentile bootstrap method. Error detection accuracy was 66.5% (95% CI: 63.6–69.1) for GPT-4.1 and 54.0% (95% CI: 50.9–57.0) for Llama 3.3 70B. Error classification accuracy was 65.4% (95% CI: 62.4–68.3) and 48.5% (95% CI: 45.4–51.8) for GPT-4.1 and Llama 3.3, respectively. Both models showed the lowest primary performance in the two-stage success rates (48.2% for GPT-4.1 and 36.0% for Llama 3.3). Primary performance metrics are shown in Table 2.

**Table 2:** Primary performance metrics with 95% CI (percentile bootstrap method)

Metric	*N* (GPT 4.1 2025-04-14)	*N* (Llama 3.3 70b)	GPT 4.1 2025-04-14	Llama 3.3 70b
Error classification accuracy	1024	1024	65.4% (95% CI: 62.4%–68.3%)	48.5% (95% CI: 45.4%–51.8%)
Error detection accuracy	1024	1024	66.5% (95% CI: 63.6%–69.1%)	54.0% (95% CI: 50.9%–57.0%)
Two-stage success rate	1024	1024	48.2% (95% CI: 45.1%–51.4%)	36.0% (95% CI: 33.1%–39.0%)

### Error type analysis

Performance differed significantly across error categories E1-E4, revealing specific knowledge gaps. E4 detection rates exceeded E2 detection rates for both GPT-4.1 (72.1–85.4% vs 33.7–47.9%, *p* < 0.001 across all modalities) and Llama 3.3 (61.5–72.9% vs 25.0–39.6%, *p* < 0.001). GPT-4.1 achieved significantly higher detection rates than Llama 3.3 across all error categories (overall: 66.5% vs 54.0%, *p* < 0.001). CT-based detection was significantly higher than MRI-based detection for E1 errors (GPT-4.1: 81.7% vs 49.0%, *p* < 0.001) and E2 errors (GPT-4.1: 46.2% vs 33.7%, *p* = 0.048).

E1 errors (anatomical mislabeling) demonstrated stark modality differences. CT showed detection rates of 81.7% (GPT-4.1) and 67.3% (Llama 3.3 70b), while MRI yielded only 49.0% (GPT-4.1) and 33.7% (Llama 3.3 70b). The overall success rate for detecting MRI E1 errors was 33.7% (GPT-4.1) and 19.2% (Llama 3.3 70b), indicating that most anatomical errors that could lead to wrong-site procedures would remain undetected.

Error category E2 (physiologically impossible findings) showed uniformly poor detection. For CT, detection rates were 46.2% (GPT-4.1) and 32.7% (Llama 3.3 70b). Performance deteriorated further for MRI (33.7% and 25.0%) and X-ray (47.9% and 39.6%). Even when detected, classification accuracy was quite low: only 35.4% of detected E2 errors in CT were correctly classified by GPT-4.1, dropping to 25.7% for MRI.

Error category E3 (diagnostic inconsistencies) achieved intermediate performance. GPT-4.1 detection rates were 88.5% (CT), 71.2% (MRI), and 75.0% (X-ray). However, classification accuracy varied significantly, particularly for X-ray, where only 55.6% of detected errors were correctly categorized.

Both models showed the highest performance for detecting E4 errors (inappropriate recommendations). GPT-4.1 achieved detection rates of 72.1% (CT), 79.8% (MRI), and 85.4% (X-ray), with excellent classification accuracy when detected (86.7%, 91.6%, and 95.1%, respectively). In comparison, Llama 3.3 70b achieved detection rates of 61.5% (CT), 67.3% (MRI), and 72.9% (X-ray), with classification accuracy of 71.9%, 80.0%, and 91.4% for detected E4 errors.

**Fig. 3:**
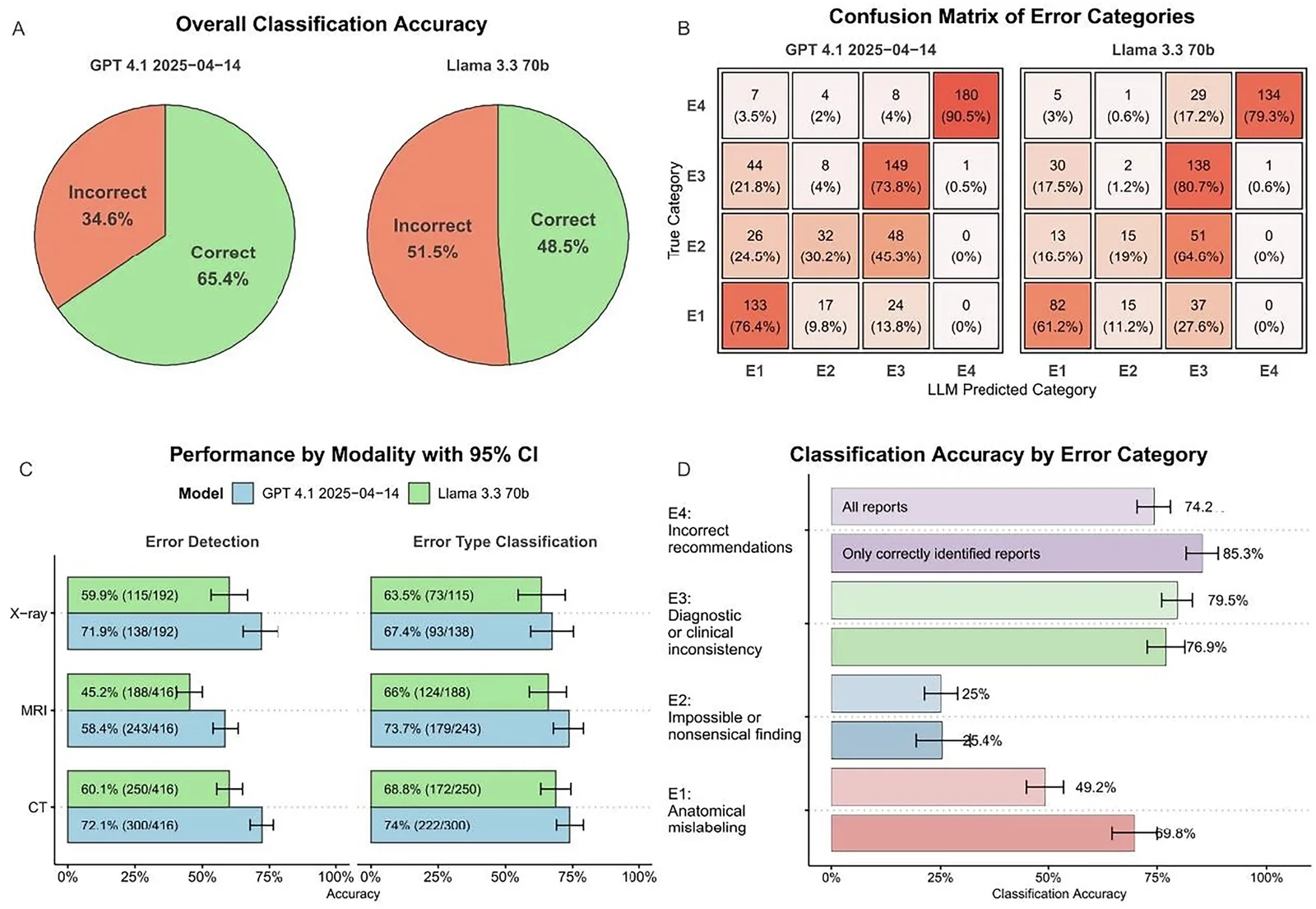
**A**–**D** Combined performance of GPT-4.1 and Llama 3.3 70b. **A** Overall classification accuracy for clinically relevant errors. **B** Confusion matrices for four error categories (E1: anatomical mislabeling; E2: physiologically impossible/nonsensical findings; E3: diagnostic/clinical inconsistencies; E4: incorrect recommendations), showing absolute numbers and percentages of true vs predicted categories. **C** Performance by imaging modality (X-ray *n* = 192, MRI *n* = 416, CT *n* = 416) for error detection and classification. **D** GPT-4.1 classification accuracy with 95% CI by error category, with lighter bars for all reports and darker bars for reports where the error was correctly detected

All performance metrics stratified by error category E1–E4 for both models are summarized in Fig. 3.

### Modality-specific performance

Model performance for detecting errors in CT reports was consistently better than for other modalities across most error categories. The performance gap was most pronounced for E1 and E2 errors. For E1 errors, the overall success rate in CT exceeded MRI by 33.6 percentage points for GPT-4.1 (67.3% vs 33.7%) and 27.0 percentage points for Llama 3.3 70b (46.2% vs 19.2%). A performance overview by imaging modality is depicted in Fig. 3C.

### Two-stage analysis

**Fig. 4:**
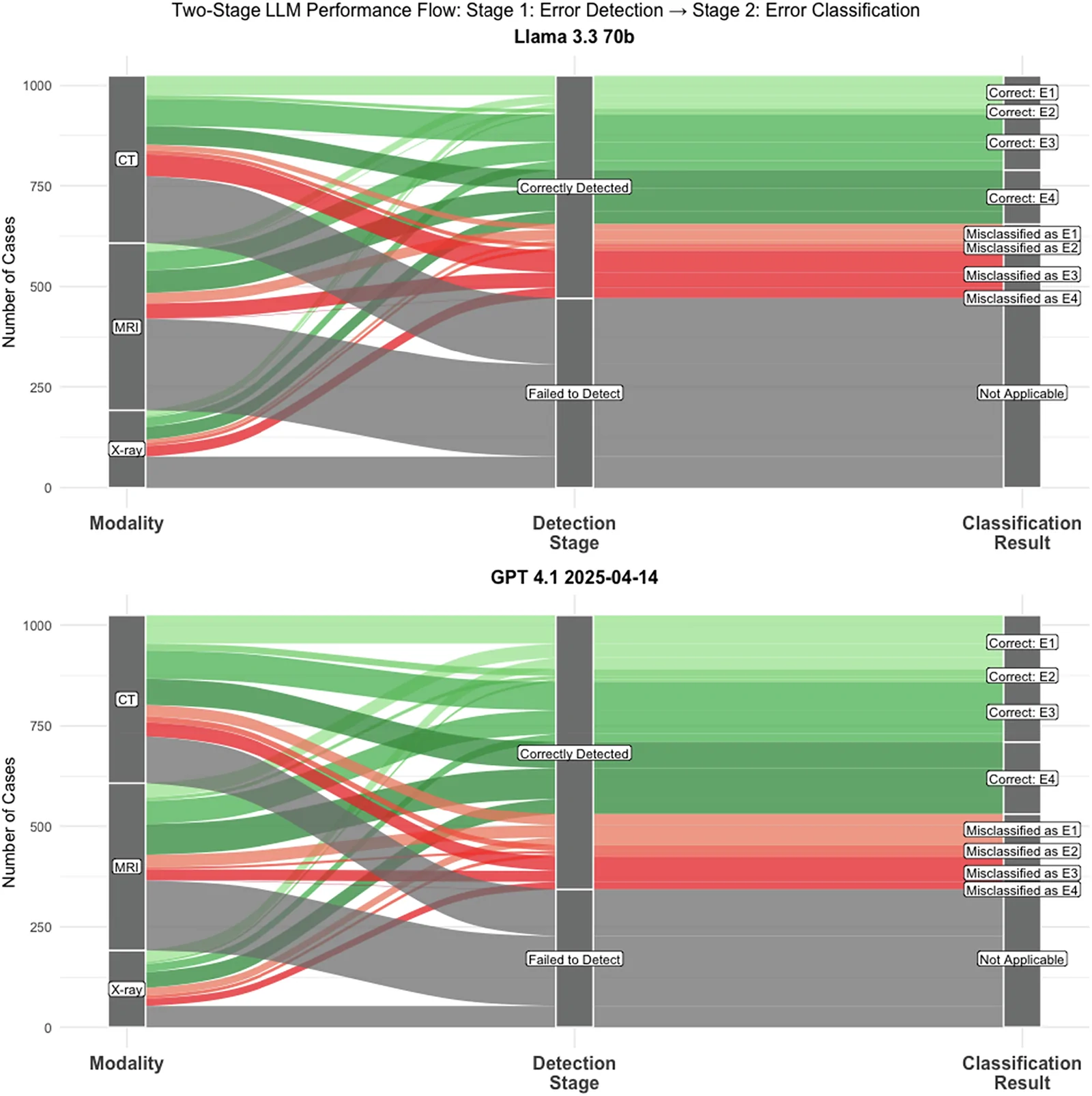
Sankey diagrams illustrating two-stage LLM performance (detection → classification) for GPT-4.1 and Llama 3.3-70b, stratified by imaging modality (CT, MRI, and X-ray)

Across all reports, both models demonstrated highly variable performance dependent on error type and modality. GPT-4.1 achieved overall success rates ranging from 8.7% to 81.2% (MRI E2 and X-ray E4, respectively). Llama 3.3 70b showed consistently lower performance with rates from 1.9% to 66.7% (MRI E2 and X-ray E4, respectively). Notably, the proportion of reports where both detection and classification were correct revealed substantial performance degradation. Even when initial detection rates appeared reasonable, poor classification accuracy resulted in low overall success rates. This was most evident for E2 errors across all modalities, where already modest detection rates were compounded by classification failures, resulting in overall success rates below 17% for GPT-4.1 and below 2% for Llama 3.3 70b. Two-stage performance is illustrated in Sankey diagrams (Fig. 4) with corresponding rates summarized in Table 3.

**Table 3:** Two-stage LLM performance metrics with 95% CI (percentile bootstrap): detection → classification for GPT-4.1 and Llama 3.3-70b across CT, MRI, and X-ray for each error type (E1–E4)

Model	Modality	Error category	*N*	Detection rate (95% CI)	Classification rate if detected (95% CI)	Overall success rate (95% CI)
GPT 4.1 2025-04-14	CT	E1	104	81.7% (74.0%–89.4%)	82.4% (74.1%–89.4%)	67.3% (58.7%–76%)
GPT 4.1 2025-04-14	CT	E2	104	46.2% (36.5%–55.8%)	35.4% (22.9%–50.0%)	16.3% (9.6%–24%)
GPT 4.1 2025-04-14	CT	E3	104	88.5% (81.7%–94.2%)	76.1% (67.4%–84.8%)	67.3% (57.7%–76%)
GPT 4.1 2025-04-14	CT	E4	104	72.1% (63.5%–80.8%)	86.7% (78.7%–93.3%)	62.5% (52.9%–71.2%)
GPT 4.1 2025-04-14	MRI	E1	104	49.0% (39.4%–58.7%)	68.6% (54.9%–80.4%)	33.7% (25%–42.3%)
GPT 4.1 2025-04-14	MRI	E2	104	33.7% (24.0%–43.3%)	25.7% (11.4%–40.0%)	8.7% (3.8%–14.4%)
GPT 4.1 2025-04-14	MRI	E3	104	71.2% (61.5%–79.8%)	79.7% (70.3%–87.8%)	56.7% (47.1%–66.3%)
GPT 4.1 2025-04-14	MRI	E4	104	79.8% (72.1%–87.5%)	91.6% (85.5%–97.6%)	73.1% (64.4%–80.8%)
GPT 4.1 2025-04-14	X-ray	E1	48	79.2% (66.7%–89.6%)	73.7% (60.5%–86.8%)	58.3% (43.8%–72.9%)
GPT 4.1 2025-04-14	X-ray	E2	48	47.9% (33.3%–62.5%)	26.1% (8.7%–43.5%)	12.5% (4.2%–22.9%)
GPT 4.1 2025-04-14	X-ray	E3	48	75.0% (62.5%–85.4%)	55.6% (38.9%–72.2%)	41.7% (27.1%–56.2%)
GPT 4.1 2025-04-14	X-ray	E4	48	85.4% (75.0%–93.8%)	95.1% (87.8%–100.0%)	81.2% (68.8%–91.7%)
Llama 3.3 70b	CT	E1	104	67.3% (57.7%–76%)	68.6% (57.1%–78.6%)	46.2% (36.5%–55.8%)
Llama 3.3 70b	CT	E2	104	32.7% (24.0%–41.3%)	26.5% (11.8%–41.2%)	8.7% (3.8%–14.4%)
Llama 3.3 70b	CT	E3	104	78.8% (71.2%–86.5%)	84.1% (76.8%–91.5%)	66.3% (57.7%–75%)
Llama 3.3 70b	CT	E4	104	61.5% (51.9%–71.2%)	71.9% (60.9%–82.8%)	44.2% (34.6%–53.8%)
Llama 3.3 70b	MRI	E1	104	33.7% (24.0%–43.3%)	57.1% (40.0%–74.3%)	19.2% (11.5%–27.9%)
Llama 3.3 70b	MRI	E2	104	25.0% (16.3%–33.7%)	7.7% (0.0%–19.2%)	1.9% (0.0%–4.8%)
Llama 3.3 70b	MRI	E3	104	54.8% (45.2%–64.4%)	80.7% (70.2%–91.2%)	44.2% (35.6%–53.8%)
Llama 3.3 70b	MRI	E4	104	67.3% (57.7%–76.0%)	80.0% (70%–88.6%)	53.8% (43.3%–63.5%)
Llama 3.3 70b	X-ray	E1	48	60.4% (45.8%–72.9%)	48.3% (31%–65.5%)	29.2% (16.7%–41.7%)
Llama 3.3 70b	X-ray	E2	48	39.6% (27.1%–54.2%)	21.1% (5.3%–42.1%)	8.3% (2.1%–16.7%)
Llama 3.3 70b	X-ray	E3	48	66.7% (52.1%–79.2%)	71.9% (56.2%–87.5%)	47.9% (35.4%–62.5%)
Llama 3.3 70b	X-ray	E4	48	72.9% (60.4%–85.4%)	91.4% (80.0%–100.0%)	66.7% (52.1%–79.2%)

## Discussion

Our systematic evaluation of GPT-4.1 and Llama 3.3 70b across 1024 error-containing radiology reports reveals a pronounced performance hierarchy governed by error type rather than overall model capability. Both models performed best on inappropriate recommendations (E4: up to 81.2% overall success) and worst on physiologically impossible findings, despite representing obvious violations of natural laws (E2: as low as 1.9%), with anatomical mislabeling (E1) and diagnostic inconsistencies (E3) falling between these extremes. However, the exact rank ordering between E3 and E4 varied by model and modality, and absolute performance levels differed substantially, with CT consistently outperforming MRI and X-ray. This pattern suggests that error type is the primary determinant of performance, with E2 errors uniformly representing the greatest challenge, while modality modulates the absolute magnitude. The consistently higher performance on CT reports may reflect the more standardized anatomical terminology and reporting conventions characteristic of CT reporting, which may align more closely with statistical patterns in the models’ training data. These results reflect the baseline capabilities of general-purpose LLMs and should not be interpreted as the upper bound of achievable performance. Domain-specific fine-tuning, prompt engineering, or hybrid architectures integrating structured medical knowledge may alter the observed performance hierarchy.

Our findings reveal a fundamental limitation in current LLMs: the inability to distinguish between linguistically plausible yet medically impossible text. With overall success rates of 8.7% (MRI) for GPT-4.1 and 1.9% (MRI) for Llama 3.3 70b, these models miss more than 90% of physiologically impossible findings. This likely reflects that LLMs operate through statistical next-token prediction [20] without encoding domain-specific knowledge, while inappropriate recommendations align more closely with formulaic training patterns [21, 22]. This distinction between linguistic plausibility and medical possibility represents a significant challenge to autonomous clinical deployment.

The performance variability of GPT and Llama across error types and imaging modalities observed in our study aligns with findings from Gertz et al, who demonstrated that GPT-4 achieved an overall detection rate comparable to human radiologists (82.7%), particularly for more surface-level errors such as spelling mistakes or omissions [23, 24]. Furthermore, as in our findings, performance declined markedly for more complex error types. Schmidt et al found that GPT-4 outperformed LLaMA-2–70b-Chat in identifying various types of clinically relevant speech recognition errors, achieving a precision of 76.9% vs 62.5% [11]. Notably, the only condition in which model performance approached published human benchmarks was GPT-4.1 on inappropriate recommendations (E4) in X-ray reports, where 81.2% overall success approximated the 82.7% detection rate reported for human radiologists by Gertz et al [23]. For all other error categories, particularly physiologically impossible findings (E2), both models fell far below expected human performance, with overall success rates as low as 1.9% (Llama 3.3, MRI E2) for errors that a radiology trainee would immediately identify.

Notably, our two-stage analysis highlights a critical limitation in current LLM-based systems: even when initial detection performance appears acceptable, poor classification drastically reduces clinical utility. Taken together, our findings and those from existing literature indicate that while GPT-4-based models can approach human-level performance in basic proofreading tasks, they fall short in reliably interpreting complex clinical content or contextual inconsistencies. Therefore, current LLMs may have value as supportive tools (for tasks like proofreading or documentation templates), but should not be deployed autonomously for clinical quality assurance without human oversight, particularly for modalities and error types where contextual reasoning is essential. These known limitations, including hallucination [25] and susceptibility to adversarial misuse [3, 14, 26], are compounded by evidence that fine-tuning (Sun et al [12]: F1 = 0.828) and prompt optimization (Schmidt et al [11]: 5–15% F1 improvement) improve detection of surface-level errors but have not been tested on the reasoning-dependent errors where our models failed most dramatically.

Both models exhibited the same performance hierarchy despite differing architectures, suggesting the pattern-recognition vs reasoning gap is a fundamental limitation of current LLM approaches rather than an artifact of model size or training data [27].

One of the limitations of this study is the use of baseline model configurations. While this allowed us to assess fundamental capabilities, it may underestimate the performance achievable through domain-specific fine-tuning or prompt engineering. As such, our findings reflect baseline diagnostic reasoning and should not be interpreted as the upper bound of model performance. Additionally, a single evaluation run was performed per model. Although the temperature was set to 0 to enforce deterministic decoding and minimize output variability, minor variance in online API outputs due to server-side implementation factors cannot be fully excluded. Repeated runs with statistical aggregation could provide more stable performance estimates, though the magnitude of the observed effect sizes (e.g., 36–69 percentage point differences in overall success between E4 and E2 errors) suggests that stochastic variation is unlikely to alter the primary conclusions. Another limitation of this study is the use of synthetic error cases. While this approach ensures consistency across test conditions, it may limit generalizability to real-world variability in radiologic reporting. However, these cases were systematically constructed and validated by board-certified radiologists to reflect plausible and clinically relevant mistakes. Moreover, a formal blinded comparison with human radiologists was not performed in this study. While two board-certified radiologists verified that all injected errors were detectable and clinically consequential, a direct human performance benchmark under conditions comparable to the LLM evaluation would provide additional context and is planned as a priority for follow-up work. Furthermore, this study focused solely on errors in radiology reports, without evaluating consistency with corresponding images. Future research should address this gap to better assess the clinical value of LLMs. Lastly, these findings are specific to GPT-4.1 and Llama 3.3 70b in their baseline, zero-shot configurations. Given the rapid pace of LLM development, newer models, fine-tuned variants, or models with retrieval-augmented generation capabilities may perform differently. The specific performance figures reported here may be superseded, while the evaluation framework and error taxonomy proposed in this study retain their value for benchmarking future models.

In conclusion, when applied to GPT-4.1 and Llama 3.3 70b in zero-shot settings, the proposed evaluation framework reveals a systematic performance hierarchy when evaluating radiology reports for clinically significant errors; up to 81.2% overall success for inappropriate recommendations that follow formulaic patterns, but as low as 1.9% for physiologically impossible findings that require domain-specific reasoning. This hierarchy persisted across CT, MRI, and X-ray modalities and across both proprietary and open-source architectures, suggesting it reflects a fundamental gap between linguistic competence and medical reasoning in current LLMs. Until this gap is addressed through domain-specific training or hybrid architectures that integrate medical knowledge, LLMs cannot be relied upon for autonomous quality assurance of radiology reports. These findings apply to general-purpose LLMs evaluated in a zero-shot setting. Domain-specific fine-tuning, prompt optimization, or hybrid architectures integrating medical knowledge bases may yield different performance profiles and should be evaluated using the framework proposed here.

## Supplementary information

ELECTRONIC SUPPLEMENTARY MATERIAL

## Data Availability

Data generated by the authors and analyzed during the study are available at: https://doi.org/10.5281/zenodo.17529445.

## Abbreviations

CI: Confidence intervals
CT: Computed tomography
LLMs: Large language models
MRI: Magnetic resonance imaging
