# Supplementary material for: GPT-4.1 and Llama 3.3 70 fail to detect clinically relevant errors in radiology reports in zero-shot evaluation
Source: Eur Radiol. 2026 Jun 19;36(11):8149–59. doi: 10.1007/s00330-026-12697-z (PMC13661699; doi:10.1007/s00330-026-12697-z)
Supplement: Supplementary file 1 [file 330_2026_12697_MOESM1_ESM.docx]

**GPT-4.1 and Llama 3.3 70 Fail to Detect Clinically Relevant Errors in Radiology Reports in Zero-Shot Evaluation**

Appendix A

# **1. Model Version Identifiers**

| **Model** | **Version Identifier** | **Provider** | **Precision** |
| --- | --- | --- | --- |
| GPT-4.1 | gpt-4.1-2025-04-14 | OpenAI | Default |
| Llama 3.3 70b Instruct | meta-llama/Llama-3.3-70b-Instruct-Turbo | Together AI | half precision (FP8) |

# **2. API Parameter Settings**

| **Parameter** | **GPT-4.1** | **Llama 3.3 70b Instruct** |
| --- | --- | --- |
| Temperature | 0 | 0 |
| Response format | JSON mode ("json_object") | JSON schema ("json_schema") |
| All other parameters | API defaults | API defaults |

# **3. System Prompt (Both Models)**

The following prompts were used in a two-stage evaluation pipeline. In Stage 1, each radiology report was assessed for the presence of factual errors. Reports flagged as containing errors were then passed to Stage 2 for error classification. The placeholder [Report] indicates where each sample radiology report was inserted.

System Prompt (used for both stages)

You are a helpful medical assistant that helps healthcare practitioners check their reports for factual errors. You will be given a random report that might contain factual errors. Your task is to first identify if the report contains any factual errors. If it does, you will then classify the error type.

Stage 1 - Error Detection Prompt

Here is a report that might contain factual errors: [Report]. Does this report contain any factual errors? Return a valid JSON object with the following structure: {“contains_factual_errors”: true/false}. Respond only with the JSON object, nothing else.

Stage 2 - Error Classification Prompt

Please analyze the following erroneous report and classify it into one of the categories: (E1) Anatomical mislabeling (e.g., “right liver lobe in left lung”). (E2) Physiologically impossible or nonsensical findings (e.g., “calcified air inclusions,” “metallic density in cerebrospinal fluid”). (E3) Subtle diagnostic or clinical inconsistencies (e.g., “benign appearance consistent with metastatic disease”). (E4) Incorrect clinical recommendations despite correct findings (e.g., inappropriate or harmful diagnostic/therapeutic suggestions based on accurately described findings). Erroneous Report: [Report]. Return a valid JSON object with the following structure: {“category”: “E1”, “justification”: “The error report contains anatomical mislabeling.”, “excerpt”: “right liver lobe in left lung”}. Return only the JSON object without any additional text or explanation. Make sure the JSON is valid and properly formatted.
